# SsPEP1, an Effector with Essential Cellular Functions in Sugarcane Smut Fungus

**DOI:** 10.3390/jof7110954

**Published:** 2021-11-11

**Authors:** Shan Lu, Yukun Wang, Xiaorui Shen, Feng Guo, Chunling Zhou, Ru Li, Baoshan Chen

**Affiliations:** 1 State Key Laboratory for Conservation and Utilization of Subtropical Agro-Bioresources and Ministry & Province Co-Sponsored Collaborative Innovation Center for Sugarcane and Sugar Industry, Nanning 530004, China; lushan@gxu.edu.cn (S.L.); 20120119@gxu.edu.cn (R.L.); 2 Guangxi Key Laboratory of Sugarcane Biology, College of Agriculture, Guangxi University, Nanning 530004, China; 3 College of Life Science and Technology, Guangxi University, Nanning 530004, China; 212020518@glmc.edu.cn (Y.W.); npdd-sq@sinocelltech.com (X.S.); 1808401003@st.gxu.edu.cn (F.G.); 1808301068@st.gxu.edu.cn (C.Z.)

**Keywords:** *Sporisorium scitamineum*, effector, cell function, pathogenicity, teliospore development

## Abstract

Biotrophic fungi have to infect their host to obtain nutrients and must establish an interaction with the host to complete their life cycle. In this process, effectors play important roles in manipulating the host’s immune system to avoid being attacked. *Sporisorium scitamineum* is the causative agent of sugarcane smut, the most important disease in sugarcane-producing regions worldwide. In this work, we functionally characterized the conserved effector PEP1 in *S. scitamineum*. The mating process and the expression of genes in the MAPK signaling pathway and the *a* and *b* loci were adversely affected in *Sspep1*-null mutants. The requirement for SsPEP1 in pathogenicity and symptom development was allele dosage-dependent, i.e., deleting one *Sspep1* allele in the mating pair turned a normal black whip with abundant teliospores into a white whip with few teliospores; however, deleting both alleles almost abolished infectivity and whip development. Δ*Sspep1* mutants produced significantly less mycelium mass within infected plants. Additionally, SsPEP1 was identified as a potent inhibitor of sugarcane POD-1a peroxidase activity, implying that SsPEP1 may function to relieve reactive oxygen species-related stress within the host plant. Taken together, our work demonstrated that SsPEP1 is a multifaceted effector essential for *S. scitamineum* growth, development, and pathogenicity.

## 1. Introduction

Smut caused by the basidiomycetous fungus *Sporisorium scitamineum* is the most important disease of sugarcane worldwide. A hallmark of the disease is the development of a black whip-like sorus (henceforth referred to as the whip or smut whip) composed of plant tissue and fungal cells in the apex of the infected sugarcane at a final stage of infection [1]. The infected sugarcane plants may tiller profusely; however, cane production is poor, resulting in the severe loss of millable stalks [2].

In the sugarcane smut fungus, two yeast-like haploid basidiospores belonging to different mating types fuse through conjugation to form a dikaryotic hyphae, which is a prerequisite for the pathogen to infect the host sugarcane and establish a systemic infection within the apical tissue. The hyphae grow in the apoplastic space between the plant cells of young apical tissue. This latent infection could be from several weeks to several months before the emergence of black whips [2,3,4,5,6,7]. In this process, a balanced biotrophic association between the host and the fungal pathogen must be reached [8,9,10]. For instance, in response to fungal infection and subsequent recognition, the host plant produces reactive oxygen species (ROS), such as superoxide anion and hydrogen peroxide (H_2_O_2_) [11,12]. Similarly, the pathogen secretes an array of proteins (effectors) into plant cells to evade recognition by the host’s immune system or disarm the host’s defenses [13,14,15,16]. In this regard, effectors encoded by the pathogen play vital roles in ensuring a successful infection [14,17,18,19]. Pep1, a secreted protein produced by the maize smut fungus *Ustilago maydis*, can reportedly inhibit the activity of an apoplastic peroxidase to prevent ROS accumulation and ultimately suppresses the oxidative burst, thereby inhibiting the pathogen-associated molecular pattern-triggered early immune response in maize [15]. In addition, *U. maydis* Pep1 and the other six proteins (Stp1 (‘stop after penetration’), Stp2, Stp3, Stp4, Stp5, and Stp6) form a stable complex that is anchored on the fungal membrane for the delivery of effectors to downregulate the host plant’s responses during plant colonization [20].

In sugarcane, ROS accumulation and the expression of antioxidant enzymes differ between the primary meristem tissues of smut-resistant and smut-susceptible sugarcane genotypes following *S. scitamineum* infection [21], while an array of inferred fungal effectors has been found to be specifically upregulated *in planta* [22,23,24]. However, little is known about the contribution of these effectors to the virulence and maintenance of the fungus within the host plant [15]. In this study, we undertook a functional characterization of a *S. scitamineum* effector SsPEP1, by knocking out the corresponding gene in one or both mating types. Our results showed that in addition to enhancing pathogenicity, SsPEP1 also had cellular functions, such as regulation of mating and teliospore development.

## 2. Materials and Methods

### 2.1. Strains, Plasmids, and Growth Conditions

*S. scitamineum* strains JG36 (mating type 1, Mat-1) and JG35 (mating type 2, Mat-2) are haploid basidiospores isolated from teliospores collected from a sugarcane whip in Guangxi, China [25]. These basidiospore strains were cultured on solid YEPS (1% yeast extract, 2% peptone, 2% sucrose) plates or in liquid YEPS medium at 28 °C [26]. The *Escherichia coli* strain DH5α (Vazyme, Nanjing, China) was grown on Luria Agar (LA) plates or Luria Broth (LB) at 37 °C. For fungus transformation, *Agrobacterium tumefaciens* strain Agl1 was grown at 28 °C on solid LA or cultured in LB [27].

The yeast strains YTK12 [28] and Y2HGold (Clontech, Beijing, China) were used for signal peptide activity verification and yeast two-hybrid interaction studies, respectively. Yeast cultures were grown in complete YPD medium (1% yeast extract, 2% peptone, 1% D-glucose) or SD-Glucose minimal medium (0.67% yeast nitrogen base, 2% D-glucose) at 30 °C with shaking at 200 rpm until reaching an OD_600_ of 0.6–0.8. 

### 2.2. Signal Peptide Verification

The *S. scitamineum Sspep1* gene was identified using the Pep1 protein of *Ustilago maydis* as the query sequence to blast the genome sequence of *S. scitamineum* strain JG36. The peroxidase gene (*pod-1a*) of sugarcane was identified using maize peroxidase-12 (POX12) as the query sequence to search the sugarcane cDNA sequences in the NCBI database. Signal peptides of SsPEP1 were predicted on the http://www.cbs.dtu.dk/services/SignalP/ (accessed on 9 October 2018) and http://www.abi.inf.untuebingen.de/Services/YLoc/ (accessed on 9 October 2018) The DNA sequences of the signal peptides were amplified by PCR with the primer pair pep1-sp-EcoRI-F/pep1-sp-XhoI-R (Table S1) using *S. scitamineum* JG36 DNA as template. The PCR products were cloned into the *Eco*RI and *Xho*I restriction sites of the pSUC2 vector using an In-Fusion Cloning Kit (TaKaRa, Beijing, China) to generate the pSUC2-Sspep1 plasmids. The constructs were transformed into yeast strain YTK12 using Yeastmaker Yeast Transformation System 2 (Clontech, Beijing, China) following the manufacturer’s protocol. 

### 2.3. Yeast Two-Hybrid Interaction Assay

The Matchmaker Gold Yeast Two-Hybrid System (Clontech, Beijing, China) was used to test the interaction between SsPEP1 and POD-1a. The *Sspep1* coding sequence (CDS) was amplified by PCR using the primer pair F-pep1-EcoRI/R-pep1-BamHI, and the *pod-1a* CDS was amplified with primer pair F-pod-EcoRI/R-pod-BamHI (Table S1). The *Sspep1* and *pod-1a* CDSs were cloned into the *Eco*RI and *Bam*HI restriction sites of pGBKT7 and pGADT7 to generate the constructs pGBKT-pep1 (bait) and pGADT-pod (prey), respectively. Yeast transformation and two-hybrid experiments were carried out following the manufacturer’s instructions. 

### 2.4. Gene Deletion in S. Scitamineum

A CRISPR/Cas9 gene-editing strategy was employed to disrupt the *Sspep1* gene using the CRISPR/Cas9/T-DNA hybrid plasmid pLS-HCas9 as a vector [27]. The target sequence (5′-ctggcggggctggcgctag-3′) of *Sspep1* was inserted between the Pu6 promotor and the sgRNA sequences by In-Fusion PCR and cloned into the *Bam*HI and *Hin*dIII restriction sites of pLS-HCas9 to yield the disruption construct pLS-pep1. *A. tumefaciens* Agl1 carrying pLS-pep1 was then used to transform *S. scitamineum* JG35 haploid cells as previously described [27]. 

### 2.5. Stress Tolerance Assay

The stress tolerance assay for *S. scitamineum* basidiospores was performed as previously described [29].

### 2.6. DNA and RNA Isolation, Reverse Transcription PCR (RT-PCR), and Reverse Transcription Quantitative Real-Time PCR (Qrt-PCR)

*S. scitamineum* strains were grown on solid YEPS plates at 28 °C for three days. DNA and RNA were extracted from fungal cells using the MiniBEST Plant Genomic DNA Extraction Kit and the MiniBEST Plant RNA Extraction Kit (TaKaRa, Beijing, China), respectively. The PrimeScript RT Reagent Kit (TaKaRa, Beijing, China) was used for cDNA synthesis. TaKaRa SYBR Premix Ex Taq II was used for real-time qPCR. The reactions were run on a LightCycler480 II. The sequences of primers used for qPCR are listed in Table S1.

### 2.7. Mating/Filamentation Assay

Haploid basidiospores of wild-type or mutant strains were grown overnight in liquid YEPS until reaching an OD_600_ of 1.0. Cells (0.5 µL) from a mixture of compatible mating types were co-spotted onto solid YEPS medium and incubated in the dark at 28 °C for 2–3 days before evaluation. 

### 2.8. Pathogenicity Assay

The pathogenicity assay was performed using the soaking inoculation method as previously described [30]. In brief, roots and bottom parts of the sugarcane tissue culture-derived plantlets were soaked into a *S. scitamineum* basidiospore cell suspension (1 × 10^6^ cells ml^−1^) and incubated at 28 °C for three days. The plantlets were planted in pots containing nursery substrate (Guiyu, Guangxi, China) and kept in a plant growth chamber at 26–28 °C with a 12-h/12-h light (2000 lux)/dark photoperiod and relative air humidity of 80–85%. The infection rate was calculated by dividing the fungal-infected plantlets (whip-showing and whip-less but with fungal mycelium in apical tissue) with total plantlets inoculated.

### 2.9. Teliospore Isolation

Teliospores in the whips of infected sugarcane plants were collected and resuspended in liquid YEPS medium for 8 h. The teliospore suspension was diluted and plated on solid YEPS medium at 28 °C for three days for single haploid colony isolation. The basidiospore mating types were identified by PCR with the primer pairs pra1F/pra1R and pra2F/pra2R specific for Mat-1 and Mat-2, respectively, as previously described [30]. 

### 2.10. Microscopy

Photographs of fungal colony morphology were taken using a Nikon SMZ25 stereomicroscope equipped with a digital camera. Sugarcane tissue samples were stained with 0.4% trypan blue following a previously described protocol [30]. Samples were visualized with an Olympus BX51 microscope operated with DP Controller software.

### 2.11. Fungal Biomass Analysis

One-centimeter-long sections from the white whips induced by the *S. scitamineum* mutants or the white portion of the black whips induced by the wild-type fungal strains were harvested for total genomic DNA isolation using the MiniBEST Plant Genomic DNA Extraction Kit (TaKaRa, Beijing, China). Relative fungal biomass quantification was expressed as copy number of the *S. scitamineum*-specific *pra1* gene per cm of whip. 

### 2.12. Peroxidase Activity Assay

The CDSs of *Sspep1* and *pod-1a* were amplified by PCR using the corresponding primer pairs pGEX-pep1-BamHI-F/pGEX-pep1-BamHI-R and pET30a-pod-BamHI-F/pET30a-pod-BamHI-R (Table S1) with cDNA generated from the whit part of a whip of infected sugarcane plants serving as a template. The fragments were cloned into the prokaryotic expression vectors pGEX4-1 and pET30a to yield the constructs pGEX4-Sspep1 and pET-Pod-1a. The plasmids were transformed into BL21 Competent Cells (Vazyme, Nanjing, China), and the expressed recombinant proteins were purified using Ni–NTA Agarose beads or GST-tag Purification Resin (both from Beyotime, Shanghai, China). In vitro horseradish peroxidase (HRP) activity was assayed by the Peroxidase (POD) Assay Kit (Solarbio, Beijing, China).

## 3. Results

### 3.1. Identification of the S. Scitamineum Sspep1 Gene

Using the sequence of the effector Pep1 from *U. maydis* [31], as a query to blast the translated nucleotide database of the *S. scitamineum* genome (https://www.ncbi.nlm.nih.gov/assembly/GCA_900002365.1, accessed on 8 March 2016), a protein with 61.3% similarity was identified, and the gene for this protein was named *Sspep1*. The coding sequence of *Sspep1* contains 531 bp and encodes a 177-amino acid protein lacking introns. A blast of the genome of JG36, a *S. scitamineum* local strain in China, confirmed the existence of Sspep1 with 100% identity to that reported (NCBI accession no. KP256757) [32].

**Figure 1:**
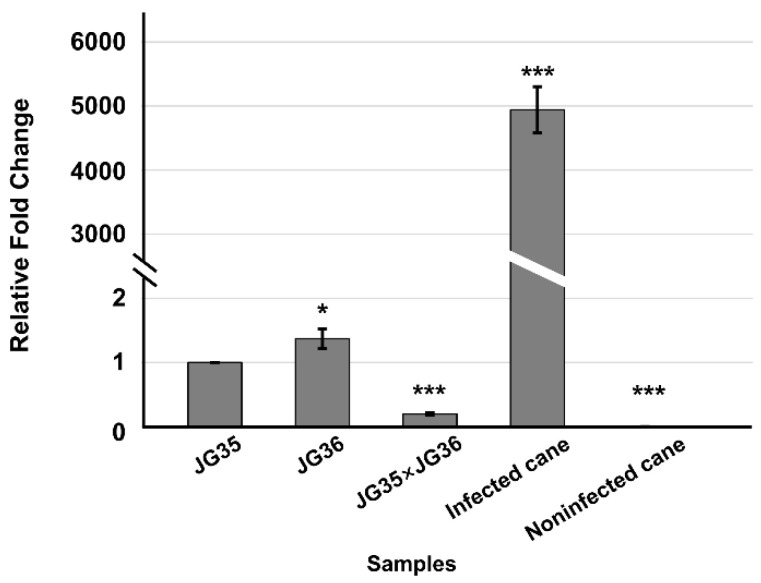
*Sspep1* transcript accumulation in vitro and in *planta*. Haploid basidiospores (JG35 and JG36) and in vitro dikaryotic hyphae (JG35 × JG36) derived from the pairing of opposite *S. scitamineum* mating types were cultured on YEPS plates for three days before being harvested for total RNA isolation. Fresh whip tissues from *S. scitamineum*-infected sugarcane plantlets were used for the isolation of total RNA. RT-qPCR was performed using cDNA reverse-transcribed from the total RNA. House-keeping gene *actin* was used as a reference gene. Transcript levels of *Sspep1* in all samples were expressed as the fold change, as compared to that of JG35, using the 2^−^^ΔΔCt^ method [33]. Means ± S.E. were derived from three independent biological repeats, each of which contained three technical repeats. *p*-values were calculated based on a Student’s t-test of replicate 2^−^^ΔΔCt^ values between a sample and JG35. * *p* < 0.05 and *** *p* < 0.001.

Comparative transcriptome analysis of in vitro haploid basidia and *in planta* infectious hyphae revealed that the expression of *Sspep1* was significantly upregulated in infected plant tissues, compared to the haploid strain (533.9:3.81, after FPKM normalization). Quantification by RT-qPCR confirmed that *Sspep1* was indeed transcriptionally induced in *in planta* upon infection with JG35 × JG36 (Figure 1). We further showed that SsPEP1 had secretion signal peptide activity. The yeast transformants carrying pSUC2-Sspep1 that contained a putative signal peptide sequence from *Sspep1* grew normally on CMD-W and YPRAA media, indicating that the enzyme sucrase fused with the SsPEP1 signal peptide could be secreted into the medium to breakdown raffinose and allow it to be used as a carbon source (Figure S1). Thus, *Sspep1* may very likely encode an effector.

### 3.2. Sspep1 Was Not Required for Basidial Growth or Stress Tolerance

The *Sspep1*-null mutant Δ35*-Sspep1* of the MAT-2 mating type strain JG35 was generated by the transformation with the CRISPR-Cas9/T-DNA vector system (Figure S2). The *Sspep1* mutant (Δ36^P^*-Sspep1*) in the opposite mating type strain JG36 was obtained by screening the basidial progeny derived from teliospores formed in the sugarcane plantlet infected with Δ35*-Sspep1* × JG36 (Figure S3). Such a mutant was marked by an uppercase P to distinguish it from the original mutant by gene disruption. No defects in cell morphology or growth rate were detected in haploid *Sspep1*-null mutants of either mating type maintained in liquid YEPS medium as compared with the wild-type strains (Figure S4), suggesting that SsPEP1 is not required for basidial growth. In stress assays on solid YEPS medium or MM-N (minimal medium minus nitrogen source) medium supplemented with Congo red (0.5 mM), SDS (0.1 mM), H_2_O_2_ (1 mM), NaCl (500 mM), or nutrient deprivation, no differences were observed between the mutants and the wild-type strains (Figure S5), suggesting that the *Sspep1* gene may not play a major role in hyperosmotic, oxidative, or cell wall integrity stress responses in *S. scitamineum*.

### 3.3. Sspep1 Deletion Mutants Displayed Attenuated Mating/Filamentation

**Figure 2:**
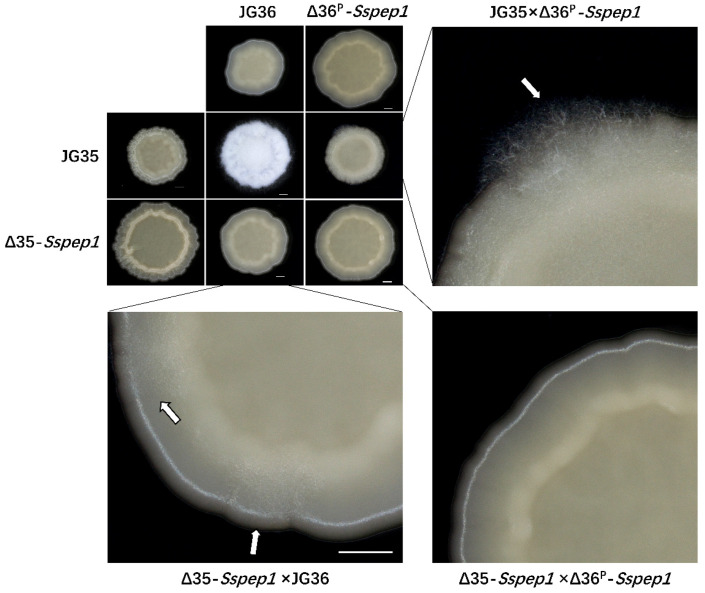
Mating behavior of *Sspep1* deletion mutants. Wild-type strains and *Sspep1* deletion mutants were co-spotted on YEPS plates and incubated at 28 °C for 72 h. White and fluffy colonies resulting from the formation of dikaryotic hyphae and filamentous growth were indicative of successful mating. Images below and on the right are enlarged colony edges showing the fluffy hyphae (arrow). Colonies were photographed under a stereomicroscope. Scale bars = 1 mm.

The co-spotting of wild-type strains JG35 × JG36 on YEPS plates resulted in mating and the formation of dikaryotic filamentous hyphae that made the colony look fluffy. However, the colonies of JG35 × Δ36^P^*-Sspep1* or Δ35*-Sspep1* × JG36 did not have the same fluffy appearance as the wild-type colonies, although hyphae could be observed in small sections of the edge of the colonies. However, hardly any hypha could be found in the Δ35*-Sspep1* × Δ36^P^*-Sspep1*-derived colonies (Figure 2). These results suggested that the mutation of *Sspep1* led to an attenuation in mating/filamentation, and the effect seemed to be *Sspep1* allele-dosage dependent.

**Figure 3:**
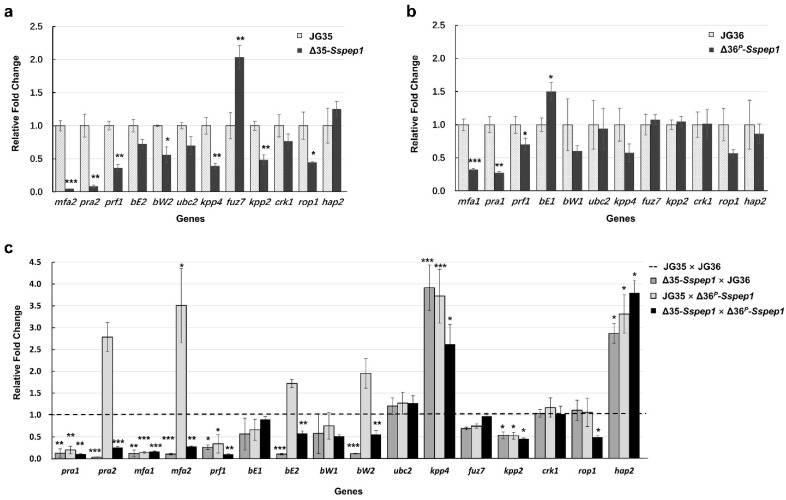
Effects of *Sspep1* deletion on the expression of genes essential for mating and filamentous growth in haploid and dikaryon stages. RT-qPCR was used to quantify the accumulation of target genes. (**a**) Δ35-*Sspep1*; (b) Δ36^P^*-Sspep1*; (**c**) mating pairs. The levels of gene expression in the wild-type haploid strains (**a**,**b**) or pairs of wild-type strains (**c**) were set as 1.0. The *actin* gene of *S. scitamineum* was used as an endogenous control. Means ± S.E. were derived from three independent biological repeats, each of which contained three technical repeats. *p*-values were calculated based on a Student’s *t*-test of replicate 2^−^^ΔΔCt^ values for each gene in the wild-type strain and *Sspep1* mutant. * *p* < 0.05, ** *p* < 0.01, and *** *p* < 0.001.

To identify the mechanism underlying this mating/filamentation regulatory effect, the expression of genes known to be involved in mating/filamentation was measured by RT-qPCR. As shown in Figure 3a, the mating factor gene *mfa2*; the pheromone receptor gene *pra2* and the pheromone response factor gene *prf1*; *bW2*, which encodes a heterodimeric transcription factor; and *kpp4*, *kpp2*, and *rop1*, key components in the MAPK signaling pathway, were all downregulated, whereas *fuz7* and *hap2*, components of the same pathway, were upregulated in haploid Δ35*-Sspep1*. However, except for *mfa1*, *pra1*, and *prf1*, which were downregulated, and *bE1*, which was upregulated, none of the genes was affected in Δ36^P^*-Sspep1* relative to that in wild-type controls (Figure 3b). These results suggested that the regulation of pheromone precursor genes, pheromone receptor genes, and pheromone response factor genes were the most likely to contribute to the defects in mating/filamentation seen in the *Sspep1*-null mutants. 

We further investigated the expression of the above-mentioned genes in mating events in which one or both *Sspep1* alleles were mutated. In Δ35*-Sspep1* × JG36, the expression of *pra1*, *pra2*, *mfa1*, *mfa2*, *prf1*, *bE2*, *bW2*, and *kpp2* was significantly downregulated, that of *kpp4* and *hap2* were upregulated, and that of *bE1*, *ubc2*, *crk1*, and *fuz7* was unchanged. In JG35 × Δ36^P^*-Sspep1*, the expression of *pra2* and *mfa2* was upregulated instead of downregulated; however, with the mating between both *Sspep1* mutants, the gene expression pattern was almost the same as that with the Δ35*-Sspep1* × JG36 mating, except that *rop1* was downregulated (Figure 3c). Combined, the results of the haploid mutants suggest that the regulation of the pheromone pathway was most likely responsible for the observed attenuation in mating and filamentation. 

### 3.4. The Deletion of Sspep1 Attenuated Virulence and Impaired Teliospore Development

**Figure 4:**
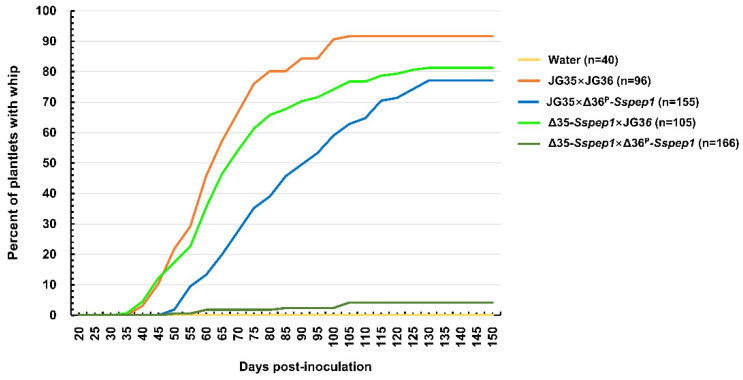
Progression of whip development induced by *Sspep1* deletion mutants. Sugarcane plantlets were inoculated with combinations of wild-type strains and/or *Sspep1*-deletion mutants at the concentration of OD = 1.0. The ‘n’ is the total number of plantlets inoculated.

To determine whether *Sspep1* is involved in the pathogenicity of *S. scitamineum*, virulence assays of mutant strains were performed on tissue culture-derived plantlets of the smut-susceptible sugarcane variety ROC22, with a total number of 96 to 166 plantlets for each assay in a plant growth chamber (Figure S6). With inoculation of the wild-type strains JG35 × JG36, 91.7% of the plantlets produced whips (WP = 91.7%) in a period of 103 days, with a mid-level whip development (MWD, when 50% of the total number of whips had developed) being attained on day 60 (MWD = 60). The figures were WP = 81.2% and MWD = 63 for JG35 × Δ36^P^*-Sspep1* and WP = 77.1% and MWD = 80 for Δ35*-Sspep1* × JG36 in a period of 127 days; and WP = 4.2% and MWD = 83 for Δ35*-Sspep1* × Δ36^P^*-Sspep1* in a period of 150 days (Figure 4). The deletion of one allele in the inoculation pair reduced the whip-producing percentage by 11.4–19.9% and led to a slight delay in whip development; however, the deletion of both alleles in the inoculation pair resulted in a marked reduction in the whip-producing percentage (91.4%), demonstrating that the effect of *Sspep1* is allele dosage-dependent.

**Figure 5:**
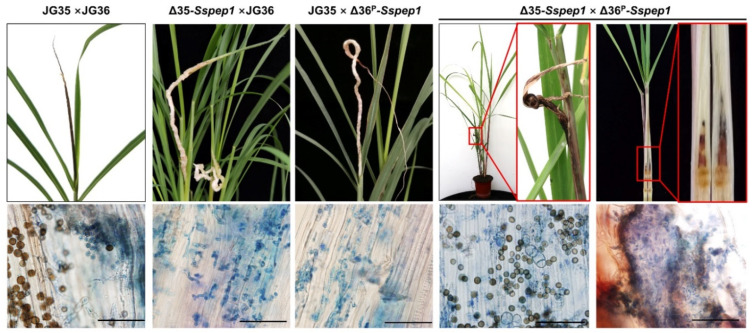
*Sspep1* deletion altered whip morphology and teliospore development. Histopathological analysis was performed on the whips or whip-less plantlets. Sections were stained with 0.4% trypan blue and viewed under a microscope. Note that no teliospores were present in white whips inoculated with Δ35*-Sspep1* × JG36 or JG35 × Δ36^P^*-Sspep1*. Tiny and hidden whips were found in plantlets inoculated with Δ35*-Sspep1* × Δ36^P^*-Sspep1*; the red boxes represent the enlarged portions of the sections. Scale bar: 200 μm.

An unexpected prominent symptom induced by the inoculation of pairs with one *Sspep1* allele deleted (Δ35*-Sspep1* × JG36 or JG35 × Δ36^P^*-Sspep1*) was the development of white whips, instead of the normal black whips seen with the wild-type pair. Inspection of the white whips revealed that no teliospores were present at the time they emerged. However, deletion of both alleles in the inoculum pair Δ35*-Sspep1* × Δ36^P^*-Sspep1* induced a few tiny black whips with seemingly normal teliospores and some apparently healthy plantlets with hidden whips (Figure 5). PCR assays confirmed that *Sspep1* was absent in these teliospores. Characterizations of plantlet phenotypes, including plantlets with normal whips, white whips, hidden whips, and those with no whips but infected with fungal hyphae, are summarized in Table 1 and Figure S7. The infection rates were 91.7% for the wild-types, 86.7% for the Δ35*-Sspep1* × JG36 pair, 82.6% for the JG35 × Δ36^P^*-Sspep1* pair, and 27.7% for the Δ35*-Sspep1* × Δ36^P^*-Sspep1* pair. 

**Figure 6:**
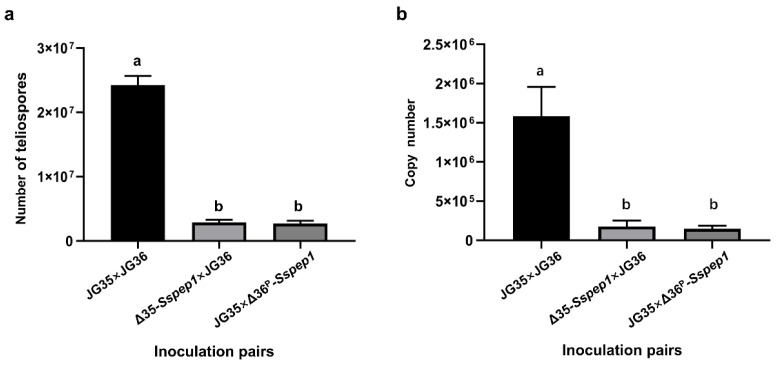
*Sspep1* deletion mutants were impaired in growth and development in the host. (**a**) Teliospores developed in infected plantlets. Data were from four whips and are expressed as numbers of teliospores per cm of whip. (**b**) Quantification of *S. scitamineum*-specific *pra1* gene copy number in the whips. The coding sequence of the *pra1* gene of *S. scitamineum* was amplified and cloned into the pMD-18 vector to generate pMD-pra1, which was used to plot the *pra1* copy number standard curve. qPCR was performed with primers pra1F01/pra1R01 (see Table S1). The copy number of the template was determined by comparing its Ct value with that of the *pra1* copy number standard curve. Apical tissue of healthy sugarcane plantlet was used as a negative control. Data were derived from three whips each with three technical replicates. Statistical differences were marked with alphabets, i.e., same letters indicate no significant difference; a and b on the columns indicate significant difference at *p* < 0.05, in a Tukey’s multiple comparison test.

To investigate whether the white whips induced by the *Sspep1* mutants would eventually form teliospores, the plants with white whips were retained and observed. After approximately 7–10 days, the whips began to darken then turned light to dark brown after 40–60 days. Teliospores were present within the whips but the numbers were only approximately 1/10 that of the wild-types (Figure 6a). Fungal DNA quantification confirmed that fungal mass in the white whips was approximately 1/9 that recorded with the wild-type fungal inoculum as determined by the DNA copy number of a specific fungal gene, *pra1*, on an equal weight basis (Figure 6b). The morphology and germination of teliospores were not seemingly affected by the deletion of *Sspep1* (Figure S8).

### 3.5. SsPEP1 Inhibited the Sugarcane Peroxidase

**Figure 7:**
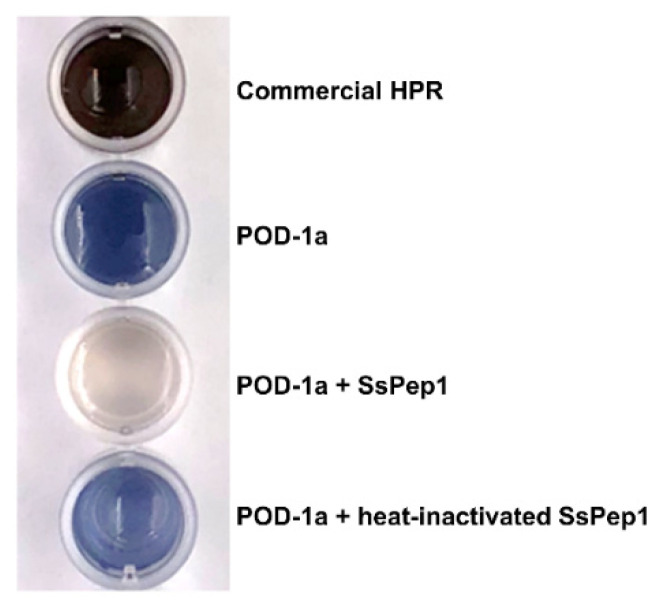
SsPEP1 potently inhibited sugarcane peroxidase activity. The substrates contained diaminobenzidine (DAB) and hydrogen peroxide (H_2_O_2_). The addition of peroxidase into the substrates led to the oxidation of H_2_O_2_ and the release of oxygen, which subsequently oxidized DAB to form a dark brown precipitate. Commercial horseradish peroxidase (HRP) served as a positive control. The peroxidase activity of POD-1a was effectively inhibited by the addition of native SsPEP1 but not heat-inactivated SsPEP1.

PEP1 of *U. maydis* was reported to interact with the peroxidase POX12 from maize and inhibit its activity, thereby reducing ROS production in the plant following infection [15]. To investigate whether SsPEP1 could interact with the peroxidase of sugarcane, we cloned the sugarcane *pod-1a* gene encoding the 331-amino-acid peroxidase-1a (POD-1a, protein ID AIY26421.1), a homolog of POX12 of maize [15]. No interaction between SsPEP1 and POD-1a was detected in a yeast two-hybrid assay (Figure S9); however, purified, prokaryotically expressed SsPEP1 could effectively inhibit the peroxidase activity of prokaryotically expressed sugarcane POD-1a in diaminobenzidine (DAB) assay in the presence of H_2_O_2_ (Figure 7). 

## 4. Discussion

Pep1 proteins are conserved in smut fungi, however, only Pep1 from *U. maydis* has been studied [15,31,32]. Pep1 was found to be secreted into the plant cell, localized in the extracellular space, interacted with POX12, a host peroxidase, and thereby suppress the ROS-mediated defensive response of the host [15,20]. *U. maydis* mutants without *pep1* could penetrate the host cell but were arrested from growing within the plant [31,32]. Very recently, Pep1 was reported to be a component of a multiprotein complex (Stp complex) that is anchored in the fungal membrane, protrudes into host cells, and likely contacts channel-forming plant plasma membrane, essential for effector delivery and virulence [20]. 

*S. scitamineum* SsPEP1 is a homolog of *U. maydis* Pep1 with 71% similarity at the amino acid level, with a functional secretion signal peptide. *Sspep1* was expressed at low levels in haploid basidia and dikaryotic hyphae in vitro, but was induced in expression during infection *in planta*, a characteristic of an effector (Figure 1). Surprisingly, *Sspep1* disruptants showed an impaired mating ability and significant downregulation of genes encoding pheromones and pheromone receptors, as well as the pheromone response factor [25,34,35] (Figure 2 and Figure 3), demonstrating that this protein plays essential roles in biological processes of the fungus. Mechanism of SsPEP1 regulation of mating in *S. scitamineum* is unknown and remains to be investigated. Previously, effector MoAa91, a homolog of the auxiliary activity family 9 protein, was reported to govern appressorium development in *Magnaporthe oryzae* [36]. 

Inoculation of sugarcane plantlets with a pair of wild-type strains (JG35 × JG36) and a wild-type and a *Sspep1* mutant combinations resulted in similar infection rates (91.6% vs. 82.6–86.7%), but only 27.7% when inoculated with a pair of two *Sspep1* mutants (Table 1). These results indicate that SsPEP1 is a qualitative factor for infection; lacking both alleles would drastically reduce the infection rate. Interestingly, white whips instead of the normal black whips were incited by the wild-type and a *Sspep1* mutant combinations (Figure 5). Given the facts that white whips were similar to the black whips in emerging time and whip size, we interpreted that hormone levels responsible for whip development in both cases may not be significantly different, i.e., half dosage of SsPEP1 does not affect the whip development. Different from the black whips, the white whips did not contain teliospores at the time of emergence, as examined by microscopy. However, these whips eventually yielded matured brown teliospores after about 40–60 days of emergence. Therefore, it is likely that the pathways for maturation and melanization of teliospores are not blocked. However, a profound difference in mycelium quantity between the black whips and the white whips (about 10:1, Figure 6) suggests a possibility that mycelium density could be a key factor contributing to the teliospore development [37]. Unexpectedly, deletion of both alleles of *Sspep1* resumed the formation of black but tiny or hidden whips (Figure 5). Since the reduction in whip number and whip size coincided with the complete deletion of SsPEP1, we speculate that SsPEP1 is required for whip development, possible by modulating the phytohormones of the host [38,39,40]. The fact that tiny and hidden whips contained normal teliospores might be explained by a quorum-sensing hypothesis [37,41,42], that mycelium density in the whips had reached the threshold for a transaction for teliospore development.

Unlike Pep1, which inhibits the ROS-inducing peroxidase activity of maize POX12 by direct interaction, SsPEP1 did not interact with POD-1a of sugarcane, a homolog of POX12, but did inhibit the peroxidase activity of POD-1a. Sugarcane POD-1a and maize peroxidase POX12 is only 37.5% (similarity 50%); the higher structures of these two proteins might also be largely different. Therefore, interaction between *U. maydis* PEP1 and POX12 may not necessarily warrant the interaction of SsPEP1 and POD-1a. Due to the unavailability of the whole genome sequence of a commercial sugarcane cultivar (Saccharum hybrid), it is currently unknown how many peroxidase isoforms exist in sugarcane. It is speculated that one of these peroxidase isoforms may or may not interact with SsPEP1. Nevertheless, the non-interaction of the two proteins does not prevent the peroxidase activity of POD-1a from being inhibited by SsPEP1. It is possible that SsPEP1 may interact with hydrogen peroxide, a substrate of peroxidase, to prevent it from oxidization by POD-1a. If proven true for other peroxidase isoforms of sugarcane, SsPEP1 may differ from *U. maydis* PEP1 in mechanism to protect the fungus from the ROS stress *in planta*.

Pep1 of *U. maydis* is secreted into the apoplastic space of maize [15]. We assume that SsPEP1 may also be delivered into the apoplastic space of sugarcane, considering its high similarity to Pep1. *S. scitamineum* systemically infects the plant by colonizing the apoplastic space in the tissue of the apex, where peroxidases of the plant are present to ensure an oxygen burst in response to fungal infection [15]. Such an environment is harsh for fungal proliferation and differentiation [21,43,44]. In this regard, effector SsPEP1, which functions as an inhibitor to suppress POD-1a-mediated ROS production, is of vital importance. While infection rates were similar, much less mycelium mass and delayed teliospore formation in plantlets infected by Δ35*-Sspep1* × JG36 or JG35 × Δ36^P^*-Sspep1* were observed, as compared with those of wild-type JG35 × JG36 (Figure 5 and Figure 6). This discrepancy could well be linked to the SsPEP1 level. The wild-type dikaryotic hyphae contain two copies of *Sspep1* and make 100% SsPEP1, while dikaryotic hyphae from wild-type and *Sspep1* mutant haploids contain only one copy of *Sspep1*, making 50% SsPEP1. Furthermore, when both alleles of *Sspep1* were deleted, the infectivity of the mutant Δ35*-Sspep1* × Δ36*^P^-Sspep1* was drastically reduced from 91.6% to 27.7% and the whip rate from 91.6% to 4%, demonstrating the essential role of SsPEP1 in guarding the mycelium from attack by the plant immunity. In this regard, *Sspep1* or SsPEP1 could serve as a target for smut management, either by an RNAi- or a plant-based antibody strategy in molecular sugarcane breeding programs.

In summary, our results demonstrated that *Sspep1* contributes to many biological processes of the fungus, including mating, filamentation, virulence, and teliospore development. Moreover, SsPEP1 was shown to be an inhibitor of peroxidase activity, implying that it plays a role in defending the fungus from ROS stress in the host plant. To the best of our knowledge, this is the first example of an effector of a sugarcane smut pathogen that exerts cellular functions important for teliospore development.

## Figures and Tables

**Table 1:** Characterization of plantlets inoculated with wild-type or mutant strains of *S. scitamineum*.

	Total Plantlets	Black Whip	White Whip	Hidden Whip (150 dpi)	Hyphae (150 dpi)
JG35 × JG36	96	88	0	0	0
Δ35-*Sspep1* × JG36	105	0	81	1	9
JG35 × Δ36^P^-*Sspep1*	155	0	126	0	2
Δ35-*Sspep1* × Δ36^P^-*Sspep1*	166	7	0	19	20

dpi: days post-infection.

## Data Availability

The data that support the findings of this study are openly available under GenBank accession number MZ497515 and MZ497516.

## Supplementary Materials

The following are available online at https://www.mdpi.com/article/10.3390/jof7110954/s1, Figure S1: Secretion assays for putative SsPep1 signal peptides, Figure S2: Construction and verification of mating-type 2 *Sspep1* deletion mutants, Figure S3: Verification of mating-type 1 *Sspep1* deletion mutants, Figure S4: Cellular structure and growth rate of Δ*Sspep1* mutants, Figure S5: Δ*SsPep1* mutants did not show defects in stress tolerance, Figure S6: A medium-scale pathogenicity assay for sugarcane smut, Figure S7: Histopathology of the whip-less plantlets, Figure S8: Morphology and germination rate of teliospores from Δ*Sspep1* mutants, Figure S9: SsPep1 did not interact with POD-1a in a yeast two-hybrid assay, Table S1: List of primers used in this study.
