# Supplementary material for: SsPEP1, an Effector with Essential Cellular Functions in Sugarcane Smut Fungus
Source: J Fungi (Basel). 2021 Nov 11;7(11):954. doi: 10.3390/jof7110954 (PMC8618092; doi:10.3390/jof7110954)
Supplement: Supplementary file 1 [file jof-07-00954-s001.zip › jof-1440169-supplementary.pdf]

Supplementary Materials:

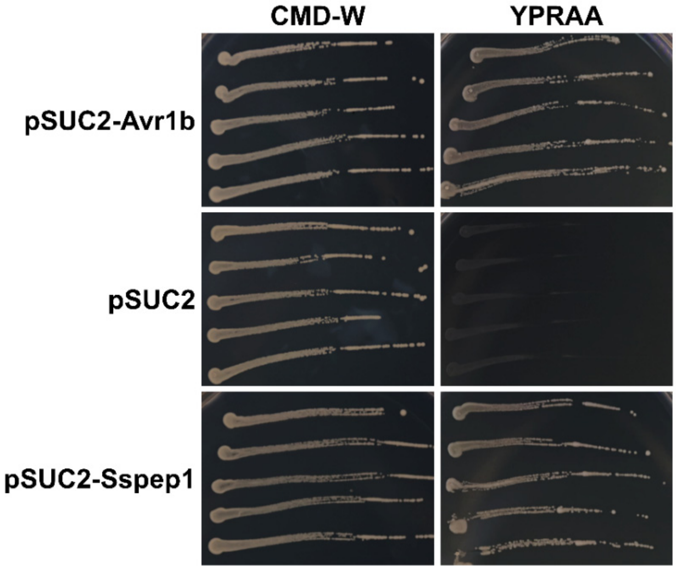

Figure S1. Secretion assays for putative SsPEP1 signal peptide. CMD-W (2% sucrose, 0.1% D-glucose, 0.075% DO supplement minus tryptophan, 2% agar) is the tryptophan-deficient medium and YPRAA (1% yeast extract, 2% peptone, 2% raffinose, 1.5% agar) is the medium with raffinose as sole carbon source. pSUC2-Avr1b served as a positive control (Avr1b is a known secretory protein) and pSUC2 as a negative control. Yeast cells transformed with the constructs were scratched on the medium plates and maintained at 30ºC for 2–3 days before observation. As seen, transformants carrying pSUC2-Sspep1, but not the vector pSUC2, survived on YPRAA medium with raffinose as the only carbon source, suggesting that the Sspep1 signal peptide-directed sucrase was able to secret into the medium to breakdown the raffinose.

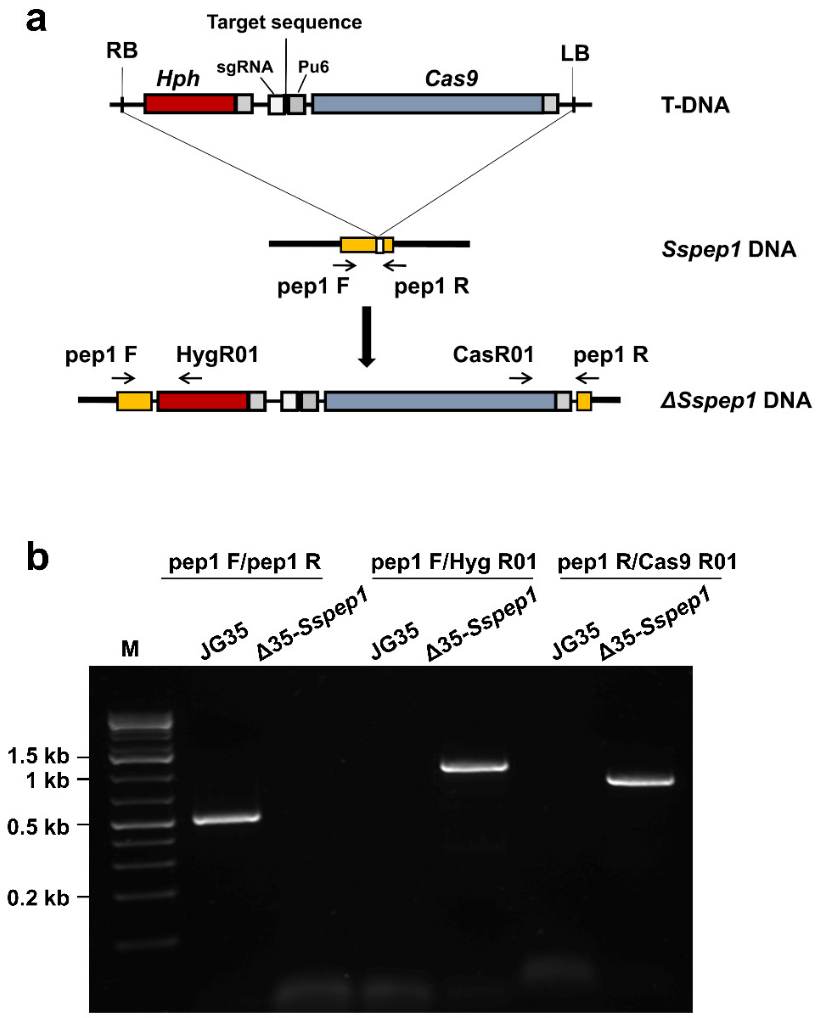

Figure S2. Construction and verification of mating-type 2 *Sspep1* deletion mutants. (**a**) Schematic representation of the *Sspep1* gene disruption strategy. (**b**) PCR verification of insertion fragments. The primer pair pep1F/pep1R was used to amplify the *Sspep1* coding region (518 bp); the primer pairs pep1F/Hyg R01 and pep1R/Cas9R01 were used to amplify the left (1,100 bp) and right (870 bp) ends of the disrupted insertion fragments, respectively.

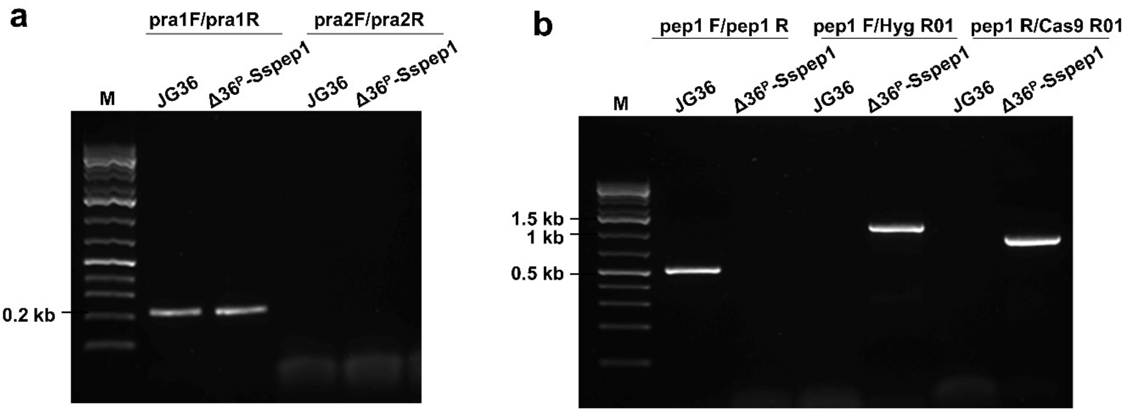

Figure S3. Verification of mating-type 1 *Sspep1* deletion mutants. (**a**) Mating type verification. The primer pair pra1F/pra1R was used to specifically amplify the *pra1* gene (221 bp) of JG36 (MAT-1) while the pra2F/pra2R primer pair was used to specifically amplify the *pra2* gene (192 bp) of JG35 (MAT-2); (**b**) PCR-based verification of the insertion fragments. The primer pair pep1F/pep1R was used to amplify the *Sspep1* coding region (518 bp) while primer pairs pep1F/Hyg R01 and pep1R/Cas9R01 were utilized to amplify the left (1,100 bp) and right (870 bp) ends of the insertion fragment of disrupted *Sspep1*, respectively.

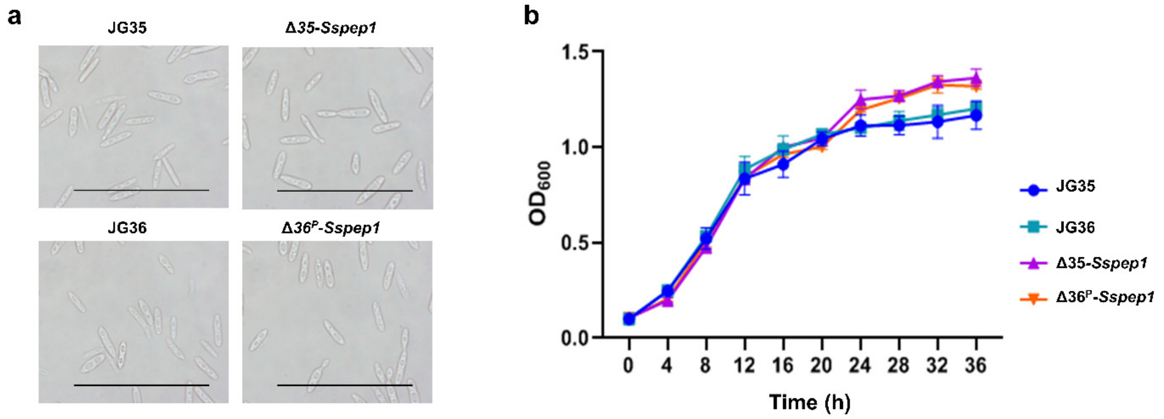

Figure S4. Cellular structure and growth rate of Δ*Sspep1* mutants. (**a**) Microscopic images of basidiospores of wild-type strains and Δ*Sspep1* mutants in two mating type backgrounds. (**b**) The growth rates of wild-type strains and Δ*Sspep1* mutants. The strains were cultured in liquid YEPS medium with an initial inoculum of 1×10^5 cells ml^{-1} at 28ºC with shaking at 200 rpm. Scale bar: 200 µm.

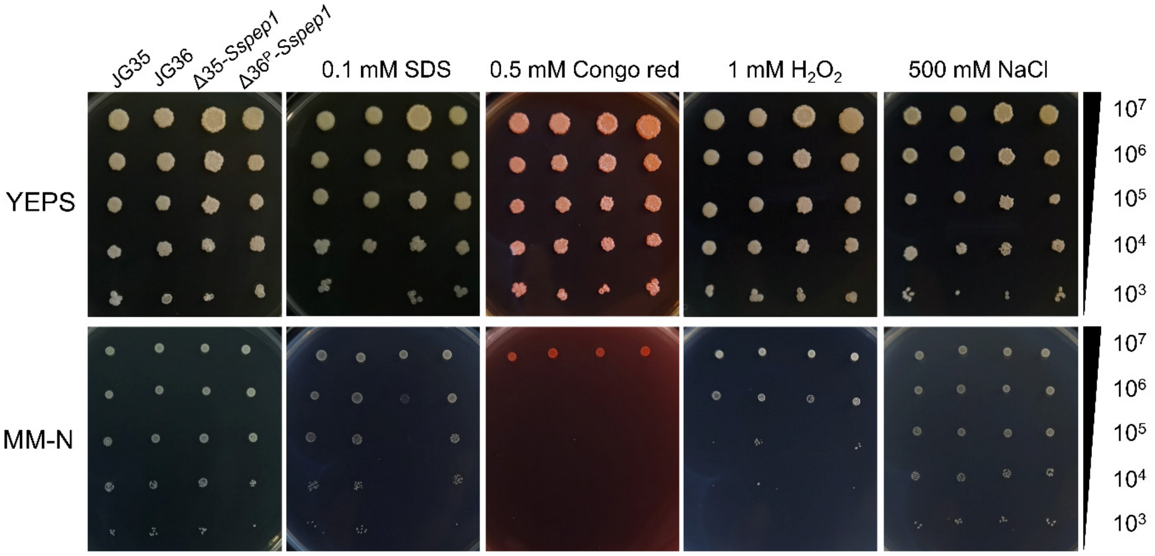

Figure S5. Δ*Sspep1* mutants did not show defects in stress tolerance. Cell concentrations are indicated at the right-most column. Test strains were spotted onto YEPS or MM-N medium supplemented with stressors and incubated at 28ºC for 72 h.

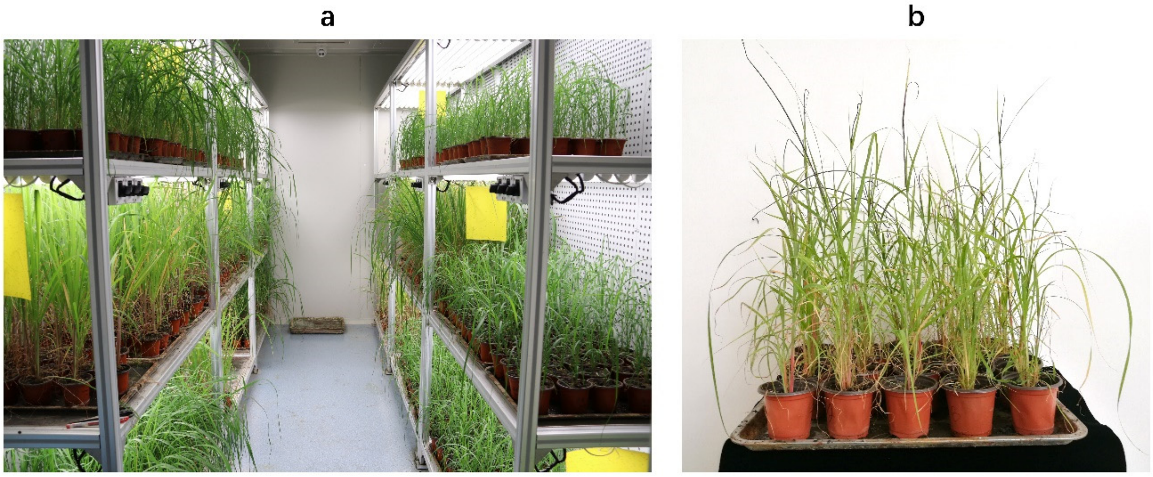

Figure S6. A medium-scale pathogenicity assay for sugarcane smut. (**a**) Pathogen inoculated tissue culture-derived plantlets were kept in a plant growth chamber with light/dark cycle of 12/12 hrs at 26-28°C. (**b**) Plantlets with whip.

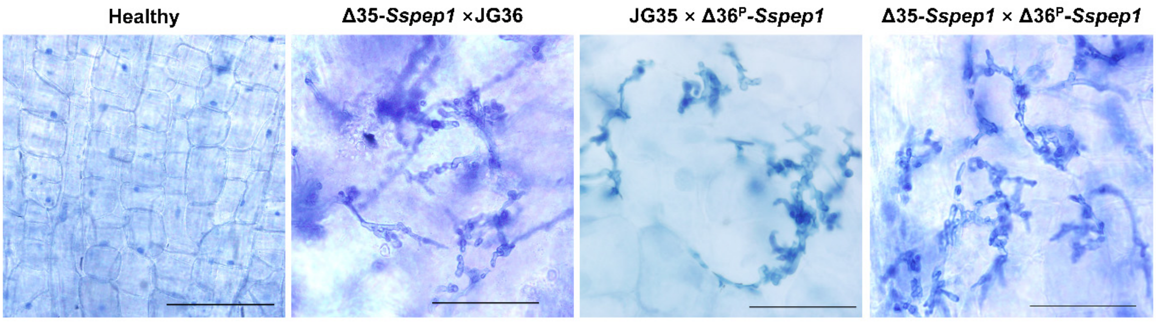

Figure S7. Histopathology of the whip-less plantlets. Dissection of meristemic tissues revealed that hyphae were present in a portion of whip-less plantlets. No hyphae were observed in the meristems of healthy sugarcanes. Scale bar, 200 µm.

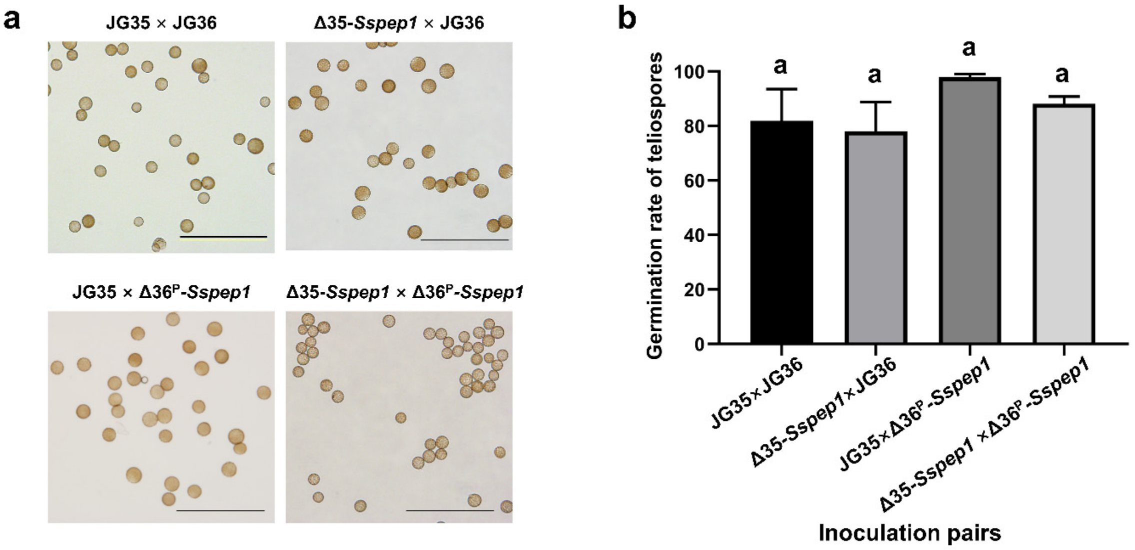

Figure S8. Morphology and germination rate of teliospores from Δ*Sspep1* mutants. (**a**) Teliospores (scale bar: 200 µm); (**b**) germination rate. Data were derived from three independent repeats.

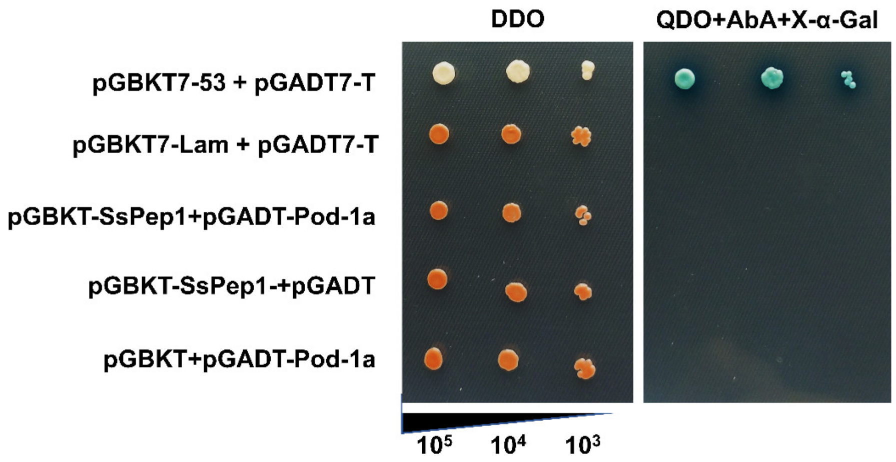

Figure S9. SsPEP1 did not interact with POD-1a in a yeast two-hybrid assay. A selection of yeast transformations were conducted on SD/-Trp-Leu (DDO) plates; selection for protein interaction was performed on SD/-Trp-Leu-His-Ade (QDO) plates supplemented with aureobasidin A (200 ng ml^{-1}) and X-α-Gal (40 μg ml^{-1}). Although transformation with all tested construct combinations allowed the yeast to grow on DDO plates in 3 days at 30°C, no construct combinations enabled the transformants to survive on QDO plates, except the positive control strain which carried plasmids pGBKT7-53 and pGADT7-T, demonstrating that SsPEP1 did not interact with POD-1a.

Table S1. List of primers used in this study

Primer	Sequence (5′–3′)
U-F	ctccgttttacctgtggaatcg
gR-R	cggaggaaaattccatccac
gRT pep1+	ctggcggggctggcgctaggttttagagctagaaatag
U6T pep1−	ctagcgccagccccgccagcgagggtaaaatctgattgtatg
gR-R	taaccatggtaccaagcttattccatccactccaagctcttg
U-Fs BamHI(in)	ctatgttactagaggatcccggaatgatctacaaagcgttcttc
Cas9 R01	ggataccgaccttccgcttcttc
HygR01	tgtatggagcagcagacgcgctac
pep1F	atgcgaaccacactgactcaag
pep1R	aagttgccgagaccgctgag
pod-1a F	atggtgacaagaatggtgctgg
pod-1a R	ctagtgcttgttgacgaaggcg
pra1F	acgtagatcgggaagaaaatgagc
pra1R	gctatctacagtggtatgtatagtc
pra2F	aagacttcaacacgattgcttttgtg
pra2R	ttgtccgaggtccgggttcccttac
Actin-For	cagctcgatgaaggtcaagat
Actin-Rev	cacatctgctggaaggtagag
pra1 F01	ccacgtaacacctttctttgcgc
pra1 R01	tcaattcgcaataacccggagc
pra2 F01	aacgcttctttcggtgttttgagc
pra2 R01	atcgcagccgaaagtccatgtc
mfa1 CDS F	atgctttccatctttacccagaccg
mfa1 CDS R	ttaggcgatggtgcagctagagtag
mfa2 CDS F	atgttcatcttcgagactgttgctg
mfa2 CDS R	ttaggccacggtgcagtagactgc
prf F01	acgtcaccgtcgacctctttcac
prf R01	ctcgcttgggaaaggagatggac
bW1 F01	atgtcgaccactgttctatctactc
bW1 R01	aatttgtgaaggtataggagtcg
bW2 F01	atgtcagcctttaactcgtccatac
bW2 R01	tacgcgtcgaggtatgcaaaactg
bE1 F01	atggcgcaacacagtagcttcgag

bE1 R01	cagcttgccgtgcaaagatggttgtc
bE2 F01	gccgaccaacagcttccgattc
bE2 R01	gcagcttgacttgcttcgaacacc
ubc2 F01	cctcacacagcccaaagtcatcg
ubc2 R01	cgagcaagacgagaacatcaccg
kpp4 F01	ctctccatgctcacggcctacaac
kpp4 R01	gaatgtcgtgttgggcaaaggtg
fuz7 F01	aagaagcgtaacttcaaagggctcc
fuz7 R01	tctggttttgatcgagagcgagc
kpp2 F01	ggcgagacgtacaagattgtcgatg
kpp2 R01	taaagtggcgcagcagcttgatc
crk1 F01	catcgtcacaaccgtctctccgac
crk1 R01	atgctgatgtggtggggaacgag
hap2 F01	acgagtctatgaaccattcatcggc
hap2 R01	agcaccaaaggaagacaagggattc
rop1 F01	tgataggggtgcaggacaaaatcg
rop1 F01	ccactcagggtgagcagagaatgc
pep1-sp-EcoRI F	ttttaattaagaattcatgcgaaccacactgactcaagc
pep1-sp-XhoI R	tagggagaacctcgaggtcggaagctttagcggaggg
pET30a-pod BamHI-F	gctcgaattcggatccatggtgacaagaatggtgctgg
pET30a-pod BamHI-R	ggctgatatcggatccctagtgcttgttgacgaaggcg
pGEX-pep1 BamHI-F	tctagaattcggatcctccgacgctataccgctgc
pGEX-pep1 BamHI-R	tggttcgcgtggatcctcacttgccaaacaagttgccg
F-pep1-EcovI	tggccatggaggccgaattcatgcgaaccacactgactcaagcc
R-pep1-BamHI	cgctgcaggtcgacggatcctcacttgccaaacaagttgccgag
F-pod-EcovI	ccatggaggccagtgaattcatggtgacaagaatggtgctggcgg
R-pod-BamHI	agctcgagctcgatggatccctagtgcttgttgacgaaggcgcag
